# Structural basis for activation and filamentation of glutaminase

**DOI:** 10.1038/s41422-023-00886-0

**Published:** 2023-10-13

**Authors:** Chen-Jun Guo, Zi-Xuan Wang, Ji-Long Liu

**Affiliations:** 1 School of Life Science and Technology, ShanghaiTech University, Shanghai, China; 2 Department of Physiology, Anatomy and Genetics, University of Oxford, Oxford, UK

**Keywords:** Cryoelectron microscopy, Molecular biology

Dear Editor,

Glutaminase catalyzes the first step of glutamine metabolism, converting glutamine to glutamate, which enters the tricarboxylic acid cycle. In human, glutaminase is encoded by two different genes, *GLS1* and *GLS2*.^1^ *GLS1* can be alternatively spliced into kidney glutaminase A (KGA) and highly active glutaminase isoform C (GAC)^1^ (Fig. 1a). GAC can be further activated by anions.^2^ It has been observed that GAC can form active filaments both in vitro and in vivo.^3,4^ Glutamine, in addition to glucose, is another vital metabolite for cell.^5,6^ Glutaminase, especially the GAC isoform, is highly overexpressed in various cancers for the utilization of glutamine.^7,8^ Currently, a specific glutaminase inhibitor is undergoing several clinical trials.^9,10^ Activating glutaminase can effectively eliminate cancer cells and solid tumors, and has significant therapeutic potentials.^11^

**Fig. 1:**
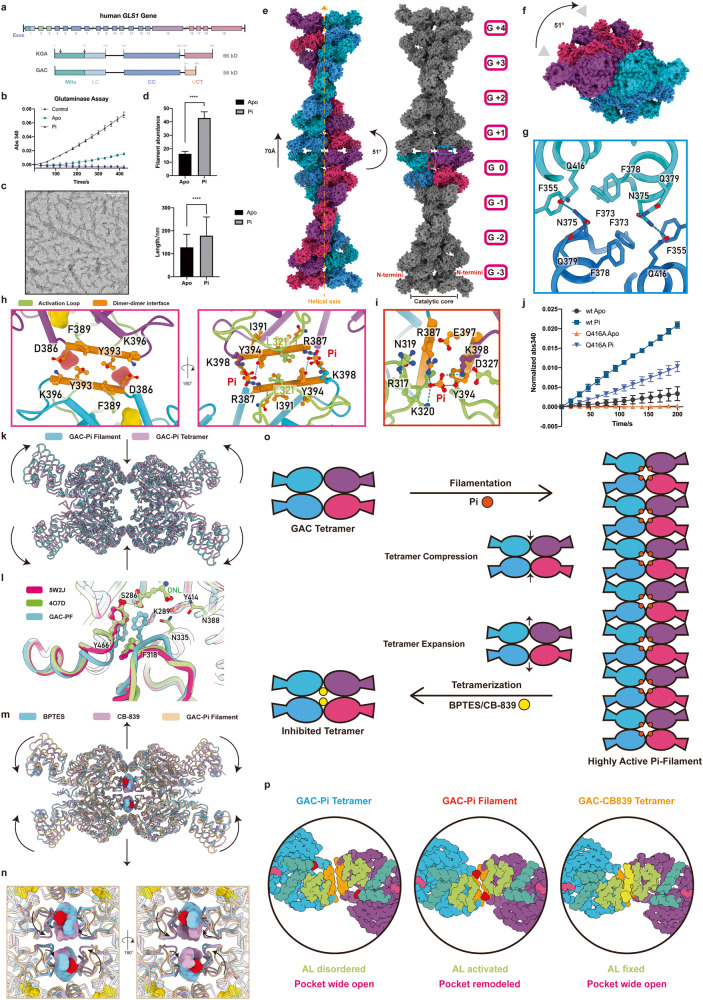
Functional and structural analysis of glutaminase filament. **a** Domain organization of KGA and GAC isoforms of human *GLS1* gene. KGA and GAC have a common N-terminal signal peptide and a catalytic core (CC) region. After mitochondrial transport, both forms lose the 72 amino acids in the head, leaving a low-complexity sequence from positions 72 to 123. The C-terminal domain of KGA consists of two ankyrin domains, while GAC has a unique C-tail without ankyrin domains. **b** Glutaminase assay for GAC^Apo^ and GAC^Pi^ with control, demonstrating the activation of glutaminase by Pi. **c** Negative-stain EM micrographs of GAC^Pi^ incubated at 37 °C for 10 min, showing the filamentation of glutaminase. Scale bar, 100 nm. **d** Quantitative analysis of negative staining images for GAC^Apo^ and GAC^Pi^, demonstrating the stimulation of glutaminase filamentation by Pi. **e** GAC-Pi filaments assembled from GAC tetramers. Rise, twist and GAC tetramer extent are indicated. The helical axis is represented by an orange dash arrow. One helical turn of the filament is displayed, consisting of ~7 helical units with each of them labeled. Left, filament colored by protomers; right, filament highlights one tetramer colored by protomers. Inside a tetramer, the protomers on the same side of the helical axis form a dimer. The interface of two dimers inside a tetramer is referred to as the “dimer–dimer interface” and is indicated by a magenta box. The helical interface and the Pi binding site are highlighted with blue and orange boxes, respectively. The catalytic core in the center of GAC filament and the N-termini on each side are annotated. **f** Top view of the GAC-Pi filament, demonstrating the helical twist in this orientation. **g** Zoom-in view of the blue box in (**e**), providing a detailed view of the interactions between GAC tetramers and the formation of the helical filament. Electrostatic interactions are indicated by the dash lines. **h** Zoom-in view of the magenta box in (**e**), demonstrating the interactions of Pi and L321 from AL with helix of 386–397. The AL, dimer–dimer interface and protomers are colored differently. **i** Zoom-in view of the red box in (**e**) for the binding site of Pi on the dimer–dimer interface, revealing the composition of the Pi binding pocket. **j** Glutaminase assay for Q416A^Apo^ and Q416A^Pi^ with GAC^Apo^ and GAC^Pi^ as control. **k** Comparison of the GAC-PF tetramer and GAC-Pi tetramer models reveals significant conformational changes. **l** The Y466 loop undergoes a conformational change in the GAC-PF tetramer upon the interaction of the reshaped AL. **m** Overall conformational changes of the GAC tetramer induced by BPTES or CB-839 binding (PDB IDs: 3UO9 and 5HL1). **n** Detailed conformational changes of AL upon binding of BPTES or CB-839 in blue and pink, respectively, highlighting the interaction between the inhibitors and AL. Pi is colored by red. The antagonistic interaction mode of Pi and inhibitors with AL is shown. **o** Agonist Pi promotes the formation of highly active GAC filaments, while the antagonists BPTES and CB-839 stimulate the formation of inactive GAC tetramers. In the highly active filament, the helical interface is located on the catalytic core of GAC, and the tetramers are compressed. When binding antagonists, GAC tetramers present an inhibited expansion mode. **p** Comparison of binding pockets from representative models. In the GAC-Pi tetramer (PDB ID: 3SS4), the AL is disordered, and the catalytic pocket is wide open. When GAC-Pi forms a filament (PDB ID: 8IMA), the AL is stabilized and the pocket is remodeled, leading to highly active GAC. When the antagonist CB-839 binds to GAC (PDB ID: 5HL1), the AL is fixed away from the pocket, leading to a wide-open pocket and GAC inhibition. AL, dimer interface, dimer–dimer interface, Pi and CB-839 are colored as follows: green, cyan, orange, red and yellow, respectively.

In 1947, it was discovered that phosphate (Pi) could significantly enhance the activity of glutaminase.^2^ To obtain a structural basis for this, GAC protein was expressed (Supplementary information, Fig. S1). The addition of Pi can stimulate both the enzymatic activity (Fig. 1b) and the filamentation of GAC (Fig. 1c, d; Supplementary information, Fig. S2). Cryo-EM samples of GAC containing Pi and an analog of glutamine, 6-Diazo-5-oxo-L-norleucine (DON) were prepared. We resolved the structure of GAC filament at a resolution of 2.9 Å (Supplementary information, Figs. S3, S4 and Table S1). GAC forms filaments with tetramers as the helical unit (Fig. 1e). By viewing the filament from the top, the twist angle of ~51° can be observed (Fig. 1f).

The helical assembly interface is composed of four protomers. The secondary structures involved in the assembly interface are α-helices 351–365, 375–384, 407–419 and β-barrel 371–373 (Fig. 1g). Sequence alignment shows that the amino acids involved in the helical interface, F355, F373, N375, F378, Q379, and Q416, are highly conserved in mammalian glutaminase (Supplementary information, Fig. S5). A GAC tetramer contains four Pi binding sites, which are located on the dimer–dimer interface, consisting of R317 and K320 on the activation loop (AL), as well as R387, Y394′ and K398′ on the dimer–dimer interface (Fig. 1h, i). Sequence alignment reveals that these amino acids are highly conserved in mammalian glutaminase (Supplementary information, Fig. S5). The Pi binding site contains multiple positively-charged amino acids. This structural feature provides a basis for the requirement of multivalent acids to fully activate GAC (Fig. 1i).

To investigate the functional significance of GAC filamentation and its relationship with Pi, we designed point mutations disrupting the filamentation interface of GAC (Supplementary information, Fig. S6a). Negative-stain electron microscopy (EM) revealed that Q416A disrupted the filamentation of GAC (Supplementary information, Fig. S6b). We then performed activity assays on Q416A. In the Apo state and the Pi bound state, the activities of Q416A are 0.26 times and 0.63 times of those of the wild-type GAC, respectively (Fig. 1j). Significant conformational changes were observed in the GAC tetramer bound with Pi when comparing the tetramers in filament to free tetramers, and the RMSD between them is greater than 2 Å. To avoid confusion, we will use the term “GAC-PF tetramer” to refer specifically to the filamentous GAC tetramer bound with Pi.

First, the GAC-PF tetramer changes into a compressed form, with each protomer rotating by 5.6° (Fig. 1k). Second, the AL (residues 308–334) of each protomer in the GAC-PF tetramer is reshaped and stabilized (Fig. 1l). The AL is a dynamic long loop involved in glutaminase reaction, being absent in most GAC structures (Supplementary information, Fig. S7). However, in the GAC-PF tetramer, the B-factor analysis of our models shows that the AL is in a stable state (Supplementary information, Fig. S8). Compared to DON-inhibited glutaminase tetramer and the dimer formed by disrupting the dimer–dimer interface (PDB codes: 4O7D and 5W2J), the AL of GAC-PF tetramer exhibits a closed conformation towards catalytic core, with the loop of 316–322 moving inward by ~2 Å (Supplementary information, Fig. S9a).

The closed AL alters the catalytic pocket of GAC (Fig. 1l). First, the stabilized AL further fills the catalytic pocket (Supplementary information, Fig. S9b, c). Second, the π–π interaction between F318 and Y466 changes the position of the Y466 loop, reducing the steric hindrance between Y466 and F318 and increasing the affinity of the catalytic pocket. This explains the decrease in enzyme activity caused by F318A reported by Sivaraman group in 2012.^12^ Finally, the downward movement of Y466, N335 and AL also reshapes the catalytic pocket (Fig. 1l). As revealed by the previous study, Y414 and Y466 act as proton transferors, while K289 acts as a proton donor (Supplementary information, Fig. S10). In the remodeled catalytic pocket, Y466, K289, and Y414 are located on the same plane, and the distance between Y466 and K289 is close, which could facilitate proton transfer and product release, thereby accelerating the reaction (Supplementary information, Fig. S9d).

CB-839 (Telaglenastat)^10^ and BPTES^13^ are non-competitive inhibitors targeting GLS1. Although both BPTES and CB-839 have fascinating specificity and anti-tumor activity, the lack of investigation of an accurate activation mechanism has, to some extent, limited our understanding of them. We aimed to examine the impact of inhibitors on GAC filament formation by adding inhibitors to pre-existing filament samples. Initially, GAC was incubated at 37 °C in a 40 mM phosphate system for 10 min, resulting in the formation of GAC filaments in the system. Upon the addition of both inhibitors, they demonstrated varying degrees of efficacy in disrupting the formation of the filaments and converting filamentous GAC tetramers into free tetramers (Supplementary information, Fig. S11a). Our quantitative analysis revealed that BPTES significantly reduced the abundance of GAC filaments and shortened the length of each filament. In contrast, CB-839 exhibited stronger ability in disassembling GAC filament and no filament was observed in the quantitatively counted images (Supplementary information, Fig. S11b, c).

The conformational changes of the tetramer explain the effect of inhibitors on GAC filament formation (Fig. 1m; Supplementary information, S12a). When BPTES or CB-839 bound to GAC, the AL interacted with inhibitors, making the outward rotation of the GAC catalytic core and the inward shrinkage of the N-termini, as compared to the GAC-PF tetramer.^13^ The inhibited tetramer exhibited a mode of expansion hindering the glutaminase filamentation. Glutaminase activity experiments conducted simultaneously with negative-stain EM demonstrated varying degrees of inhibition of GAC activity by BPTES and CB-839. Specifically, at a concentration of 0.9 μM, BPTES exhibited significant inhibition of GAC activity, while CB-839 almost completely inhibited GAC activity (Supplementary information, Fig. S11d).

Comparing GAC-PF tetramer with inhibitor-bound tetramer, we find that the natural activator Pi and inhibitors are antagonistic competitive ligands of GAC. On the one hand, both Pi and inhibitors bind to the dimer–dimer interface. When two tetramers are aligned, the binding of inhibitors and Pi overlaps with each other; thus the binding of inhibitors would create steric hindrance and directly limit Pi binding and GAC activation (Fig. 1n; Supplementary information, S12b). The overlap of the binding sites also suggests that Pi and inhibitors cannot co-exist in GAC. On the other hand, compared to the ordered fixation of the AL by Pi, inhibitor binding fixes the AL in a different mode, keeping it away from the catalytic active site and making the catalytic reaction less likely to occur (Fig. 1n; Supplementary information, S12b).

In this study, utilizing cryo-EM and available structures, we determine the binding site of Pi and propose a mode and mechanism of GAC activity regulation by different ligands and GAC filamentation (Fig. 1o). In the GAC-Pi tetramer, the AL is disordered, and the catalytic pocket is wide open. However, when Pi-bound GAC forms a filament, the AL is stabilized, and the pocket is remodeled, resulting in highly active GAC. When the antagonist CB-839 binds to GAC, the AL is fixed away from the pocket, resulting in a wide-open pocket and inhibition of GAC (Fig. 1p).

Our work provides the long-sought activation mechanism of glutaminase, and significantly advances our understanding of the inhibition mechanism and filamentation of glutaminase.

### Supplementary information

Supplementary information

## Data Availability

The structure data are available under EMD accession codes EMD-35573 and EMD-35574, and PDB codes 8IMA and 8IMB.
