# Supplementary material for: Structural basis for activation and filamentation of glutaminase
Source: Cell Res. 2023 Oct 13;34(1):76–9. doi: 10.1038/s41422-023-00886-0 (PMC10770349; doi:10.1038/s41422-023-00886-0)
Supplement: Supplementary file 1 — Supplementary information [file 41422_2023_886_MOESM1_ESM.pdf]

Methods

GAC plasmid construction and protein purification

The human GAC (amino acids 123-598) gene was cloned into pET28a vector with a N-terminal 6 × His SUMO[1] tag and transformed into Escherichia coli Transetta (DE3) cells for expression. Transformed cells were cultured in LB medium at 37 °C with 220 rpm until OD600 reached a range of 0.4 to 0.6,and then down-tempered to 18 °C for 0.5 to 1 hr before induction with 0.1 mM IPTG for 16-20 hr at 18 °C. Cells were pelleted by centrifugation at 4,500 rpm for 18 min followed by resuspension in cold lysis buffer (50 mM Tris-HCl pH 8.5, 500 mM NaCl, 10% glycerol, 20mM imidazole, 1 mM PMSF, 5mM β-mercaptoethanol, 5mM benzamidine, 2 μg/ml leupeptin, and 2 μg/ml pepstatin). Bacteria in lysis buffer were disrupted by high pressure at 750 bar and centrifuged at 18,000 rpm for 1 hr at 4 °C to collect supernatant. This was incubated with equilibrated Ni-Agarose (Qiagen) for 1 hr. Next, Ni-Agarose was washed by washing buffer (50 mM Tris-HCl pH 8.5, 500 mM NaCl, 10% glycerol, 40mM imidazole), and proteins were eluted with elution buffer (30 mM Tris-HCl pH 8.5, 100 mM NaCl, 240 mM imidazole,5 mM β-mercaptoethanol), peak fractions were treated with SUMO protease ULP1 for 1 hr at 8 °C. SuperoseTM 6 Increase 10/30 GL column and AKTA Pure (Cytiva) were used for further purification. Finally, GAC was eluted with buffer

containing 100 mM NaCl and 30 mM Tris-HCl pH 8.5.

Glutaminase Assay

GAC activity was determined using the L-Glutamate Dehydrogenase-based (GDH), two-step Glutaminase protocol as previously published[2]. GAC was incubated in the reaction buffer containing 30 mM Tris-HCl pH 8.5, 10 units GDH and 2 mM NAD+ for 5-10 min at room temperature or 37 °C. For inhibitor assay, 0.9 μM inhibitors, which were dissolved by DMSO, were added to the system, and then incubated at 37 °C for 10 min. To initiate reaction, 50 mM glutamine was added into the mixture. Absorption of a wavelength of 340 nm of each reaction mixture was measured with SpectraMax i3 as the indication for NADH levels at individual time points and absorbance represents the glutaminase activity.

Negative Staining

13 μM GAC was incubated for 10 min at 37 °C for preparation of GACApo sample. In other cases, GAC proteins were first incubated with 40 mM Pi for 10 min at 37 °C, and then mixed with inhibitors with concentration of 0.9 μM for 10 min at 37 °C. The prepared protein samples were applied to glow-discharged carbon-coated EM grids (400 mech, EMCN), and stained with 1% uranyl formate. Images were acquired at 57,000× magnification using a Tecnai Spirit G21 microscope (FEI).

Cryo-EM Grid Preparation and Data Collection

For preparing the Pi-bound GAC sample, 12 μM GAC was first incubated with 2.3 mM DON for 30 min at 37 °C, and then incubated with 40 mM Pi for 5 min at 37 °C. Samples were prepared with 100 holey carbon film (Q46208-Cu200-R0.6/1) and FEI Vitrobot (4 °C temperature, 3.5 s blotting time, −1 blot force). Images were taken with a Gatan K3 summit camera on a FEI Titan Krios electron microscope operated at 300 kV. The magnification was 22,500 × in superresolution mode with the defocus rage -1.2 to -1.8 μm and a pixel size of 1.06 Å. The total dose was 50e−/Å2 subdivided into 40 frames at 2.8-s exposure using SerialEM.

Image Processing

The whole workflow was done in RELION 3.1.2. The single-particle analysis strategy was employed throughout the entire processing. Raw movies were dose weighted and aligned by MOTIONCOR2 through RELION3 GUI, and contrast transfer function (CTF) parameters were determined by CTFFIND4. 3,271,231 particles were picked by autopicking. After two-dimensional and three-dimensional (3D) classification with C1 and D2 symmetry, 611,365 particles were selected for the 3D refinement. 321,414 particles centering on interface generated a map of 3.0 Å, and 203,004 particles centering on tetramer generated a map

of 3.1 Å. CTF refinement and Bayesian polishing were applied to each particle. The 3D refinement and continued focus refinement with tight mask were used in two different particle sets. Finally, we constructed maps for GAC-Pi filament both centering on the helical interface and helical unit. The overall gold-standard resolution calculated at the FSC (FSC = 0.143) was 2.90 and 2.86 Å. The map centering on the helical interface was used to estimate the helical parameters of GAC-Pi filament.

Model Building and Refinement

Previous model [Protein Data Bank (PDB) ID:3SS4] was applied for the initial model. Model of monomer was manually refined according to the electron density map with Coot software[3]. The refined monomer model was symmetrized to build tetramer models in Chimera software[4]. The tetramer models were subsequently real-space refined in Python-based hierarchical environment for integrated xtallography (Phenix) software[5].

Quantification and statistical analysis

Images of negative staining is processed using ImageJ[6] for quantification. Polymers of length greater than 50 nm were considered as filaments. Results of quantification and glutaminase assay were analyzed using GraphPad Prism 8[7] and were shown as means ± SD of three or more independent experiments. Mafft program[8] was used for alignment of

sequence of human KGA and GAC (UniProtKB:O94925;homo sapiens), human GLSL (UniProtKB:Q9UI32;homo sapiens), rat GLSK (UniProtKB:P13264;Rattus norvegicus), rat GLSL (UniProtKB:P28492;Rattus norvegicus), mouse GLSK (UniProtKB:D3Z7P3;Mus musculus), mouse GLSL (UniProtKB:Q571F8;Mus musculus), lion GLS (UniProtKB:A0A8C8XW40,Panthera leo), pig GLS (UniProtKB:A0A4X1THT5,Sus scrofa), and goat GLS (UniProtKB:A0A452DX27,Capra hircus). The result of sequence alignment was visualized by ESPript 3[9] which rendered sequence similarities and structure information taking crystal structure of dimeric form of mouse Glutaminase C (PDB EntryID:5W2J) as reference.

Reference

1 Johnson, E. S. Protein modification by SUMO. *Annual review of biochemistry* **73**, 355-382 (2004).

2 Kenny, J. *et al.* Bacterial expression, purification, and characterization of rat kidney-type mitochondrial glutaminase. *Protein Expr Purif* **31**, 140-148 (2003). https://doi.org:10.1016/s1046-5928(03)00161-x

3 DiMaio, F. & Chiu, W. Tools for Model Building and Optimization into Near-Atomic Resolution Electron Cryo-Microscopy Density Maps. *Methods Enzymol* **579**, 255-276 (2016). https://doi.org:10.1016/bs.mie.2016.06.003

4 Pettersen, E. F. *et al.* UCSF Chimera--a visualization system for exploratory research and analysis. *J Comput Chem* **25**, 1605-1612 (2004). https://doi.org:10.1002/jcc.20084

5 Adams, P. D. *et al.* PHENIX: building new software for automated crystallographic structure determination. *Acta Crystallogr D Biol Crystallogr* **58**, 1948-1954 (2002). https://doi.org:10.1107/s0907444902016657

6 Abràmoff, M. D., Magalhães, P. J. & Ram, S. J. Image processing with ImageJ. *Biophotonics international* **11**, 36-42 (2004).

7 Swift, M. L. GraphPad prism, data analysis, and scientific graphing. *Journal of chemical information and computer sciences* **37**, 411-412 (1997).

8 Katoh, K., Misawa, K., Kuma, K. i. & Miyata, T. MAFFT: a novel method for rapid multiple sequence alignment based on fast Fourier transform. *Nucleic acids research* **30**, 3059-3066 (2002).

9 Robert, X. & Gouet, P. Deciphering key features in protein structures with the new ENDscript server. *Nucleic acids research* **42**, W320-W324 (2014).

1. Johnson, E.S., *Protein modification by SUMO.* Annual review of biochemistry, 2004. **73**(1): p. 355-382.
2. Kenny, J., et al., *Bacterial expression, purification, and characterization of rat kidney-type mitochondrial glutaminase.* Protein Expr Purif, 2003. **31**(1): p. 140-8.
3. DiMaio, F. and W. Chiu, *Tools for Model Building and Optimization into Near-Atomic Resolution Electron Cryo-Microscopy Density Maps.* Methods Enzymol, 2016. **579**: p. 255-76.
4. Pettersen, E.F., et al., *UCSF Chimera--a visualization system for exploratory research and analysis.* J Comput Chem, 2004. **25**(13): p. 1605-12.
5. Adams, P.D., et al., *PHENIX: building new software for automated crystallographic structure determination.* Acta Crystallogr D Biol Crystallogr, 2002. **58**(Pt 11): p. 1948-54.
6. Abràmoff, M.D., P.J. Magalhães, and S.J. Ram, *Image processing with ImageJ.* Biophotonics international, 2004. **11**(7): p. 36-42.
7. Swift, M.L., *GraphPad prism, data analysis, and scientific graphing.* Journal of chemical information and computer sciences, 1997. **37**(2): p. 411-412.
8. Katoh, K., et al., *MAFFT: a novel method for rapid multiple sequence alignment based on fast Fourier transform.* Nucleic acids research, 2002. **30**(14): p. 3059-3066.
9. Robert, X. and P. Gouet, *Deciphering key features in protein structures with the new ENDscript server.* Nucleic acids research, 2014. **42**(W1): p. W320-W324.

Supplementary figures S1-S12

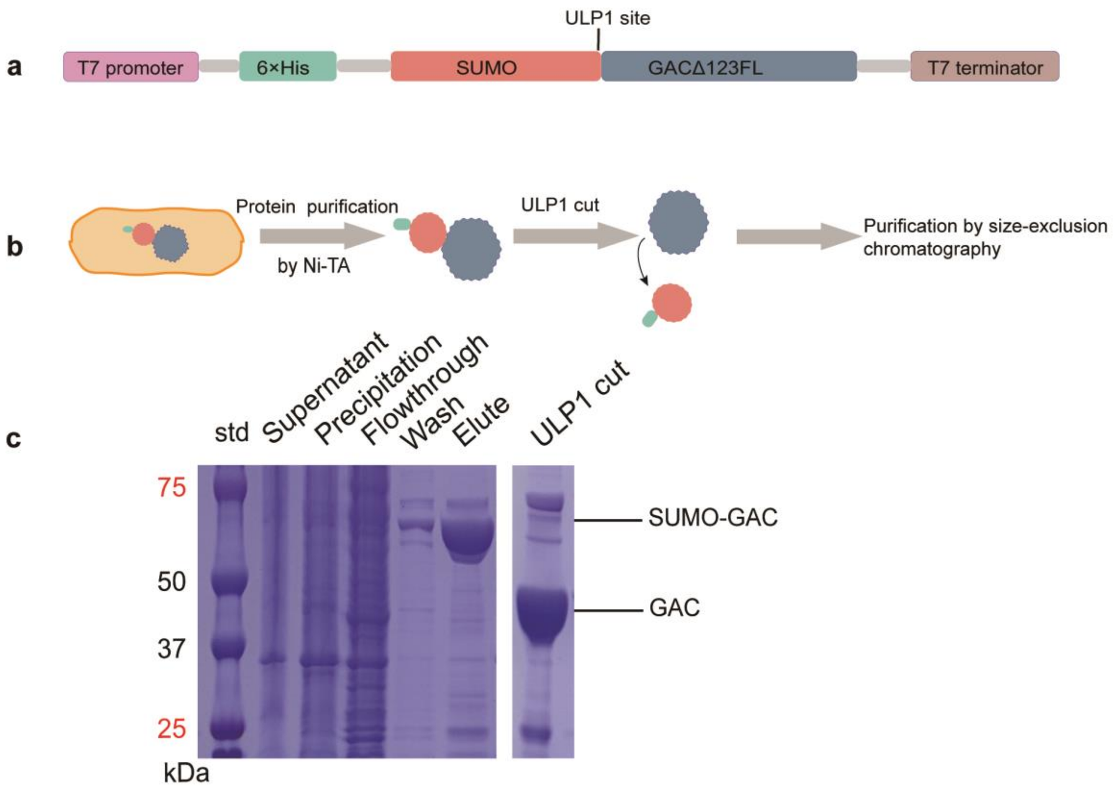

Fig. S1. GAC plasmid construction and protein purification.

a) Plasmid construction. The plasmid was designed for the expression of hGAC with a 6×His SUMO tag at the N-terminus. **b)** Protein purification of hGAC by Ni-NTA. The 6×His SUMO tag was cleaved by ULP1, followed by further purification by size-exclusion chromatography. **c)** SDS-Page analysis of purified hGAC proteins showing the cleavage of SUMO tag.

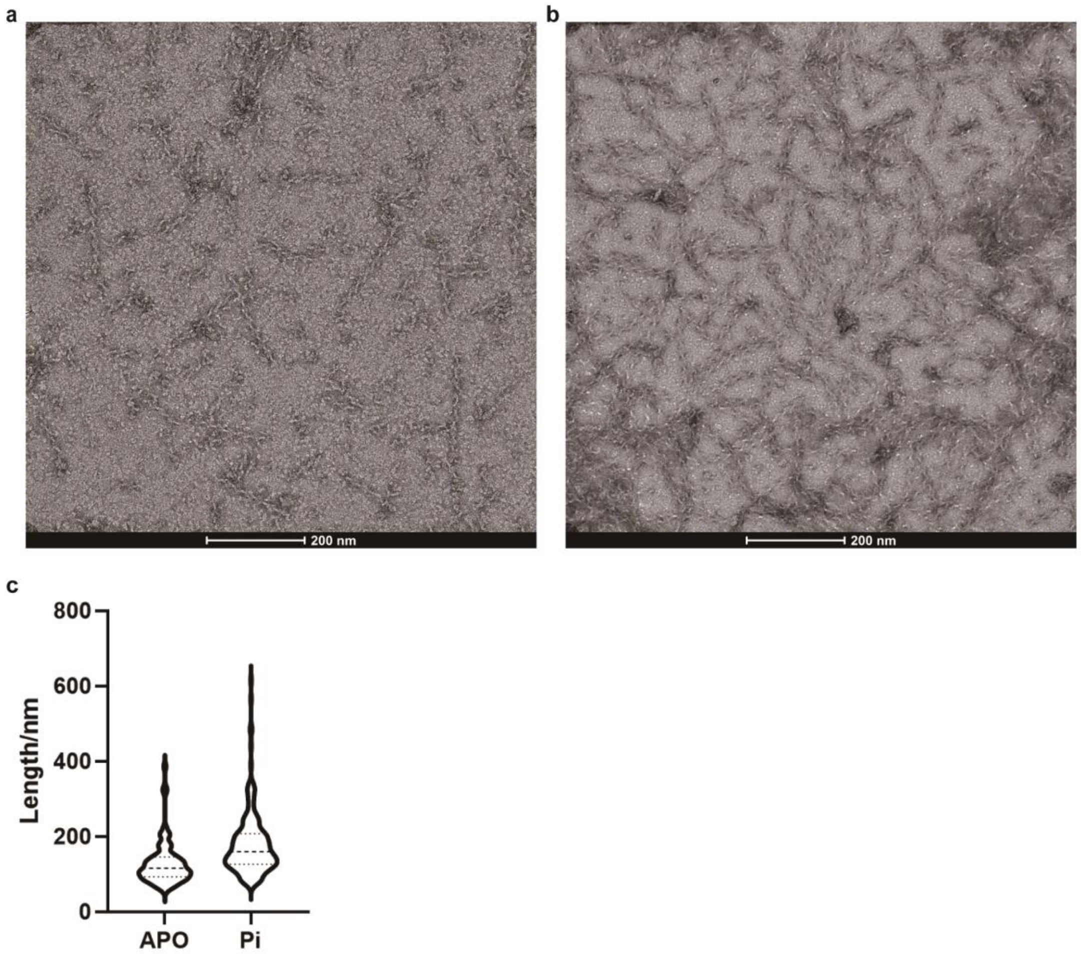

Fig. S2. Phosphate stimulates the filamentation of GAC.

Negative stain electron microscopy micrographs of GAC incubated at 37 °C for 10 min, showing the filamentation of glutaminase. The scale bar is defined in the graph as 200 nm. **a)** GAC^{Apo}. **b)** GAC^{Pi}. **c)** The distribution of filament length.

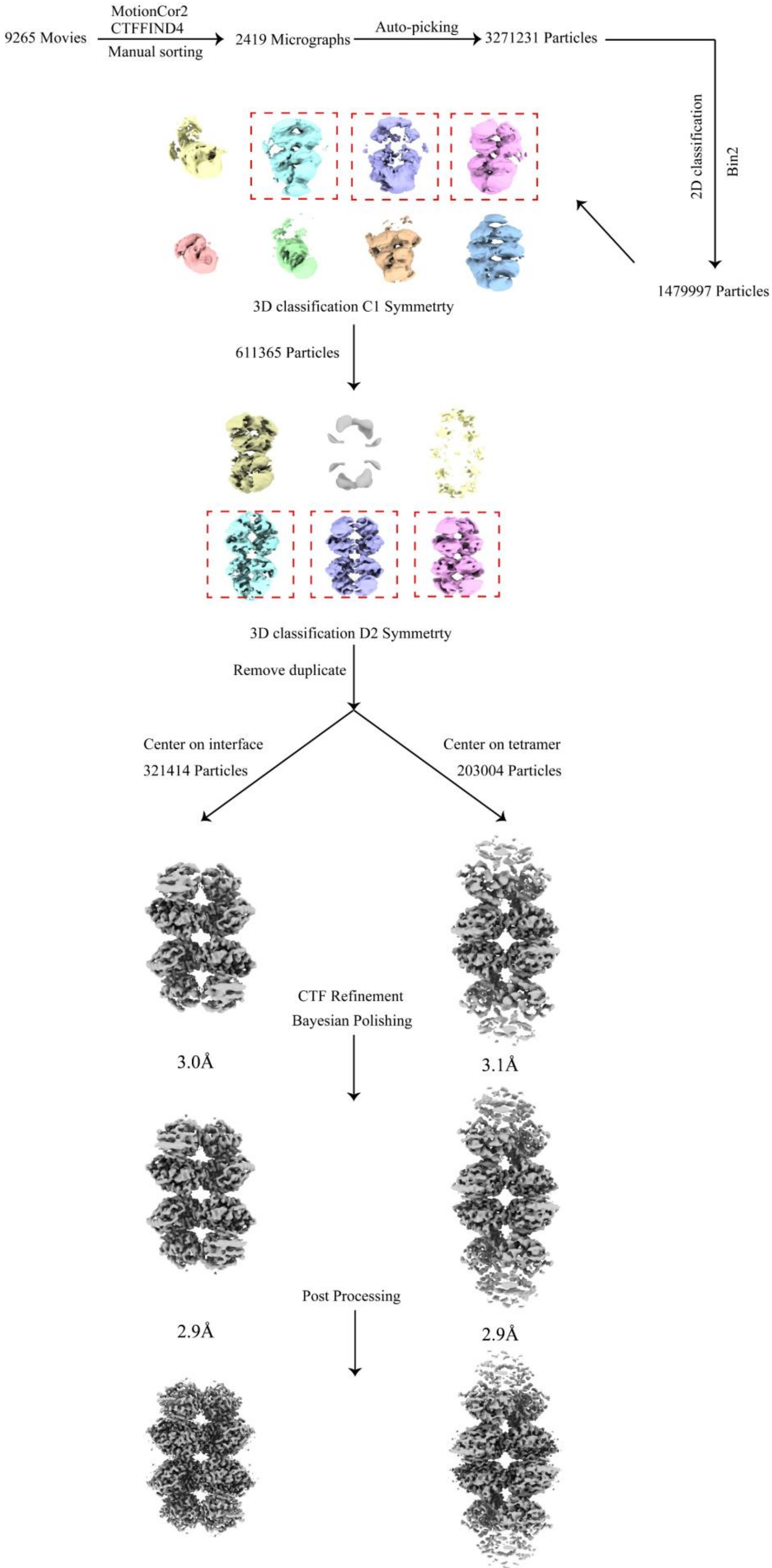

9265 Movies
MotionCor2
CTFFIND4
Manual sorting
2419 Micrographs
Auto-picking
3271231 Particles
2D classification
Bin2
1479997 Particles
3D classification C1 Symmetrty
611365 Particles
3D classification D2 Symmetrty
Remove duplicate
Center on interface
321414 Particles
Center on tetramer
203004 Particles
3.0Å
3.1Å
CTF Refinement
Bayesian Polishing
2.9Å
2.9Å
Post Processing

Fig. S3. The workflow of data processing.

After initial particle picking and 2D classification, 3D classification was performed to select well-aligned filaments. Focused refinement was then carried out on both the helical unit and helical interface to improve the resolution of the final structures. Further details can be found in the Method section.

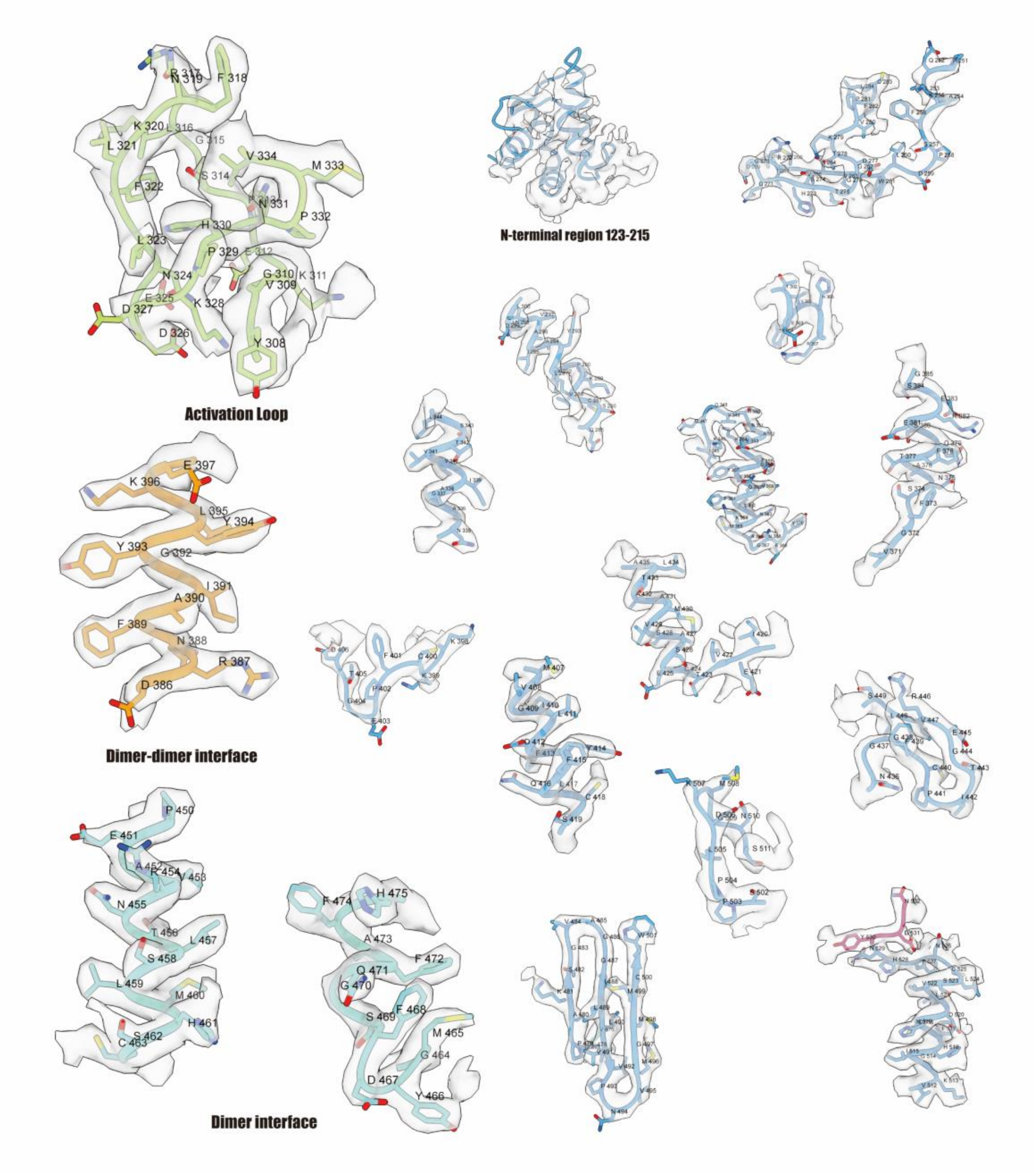

Fig. S4. Representative EM detail.

The transparent surface represents the density map, and the amino acid residues are indicated with single-letter abbreviations.

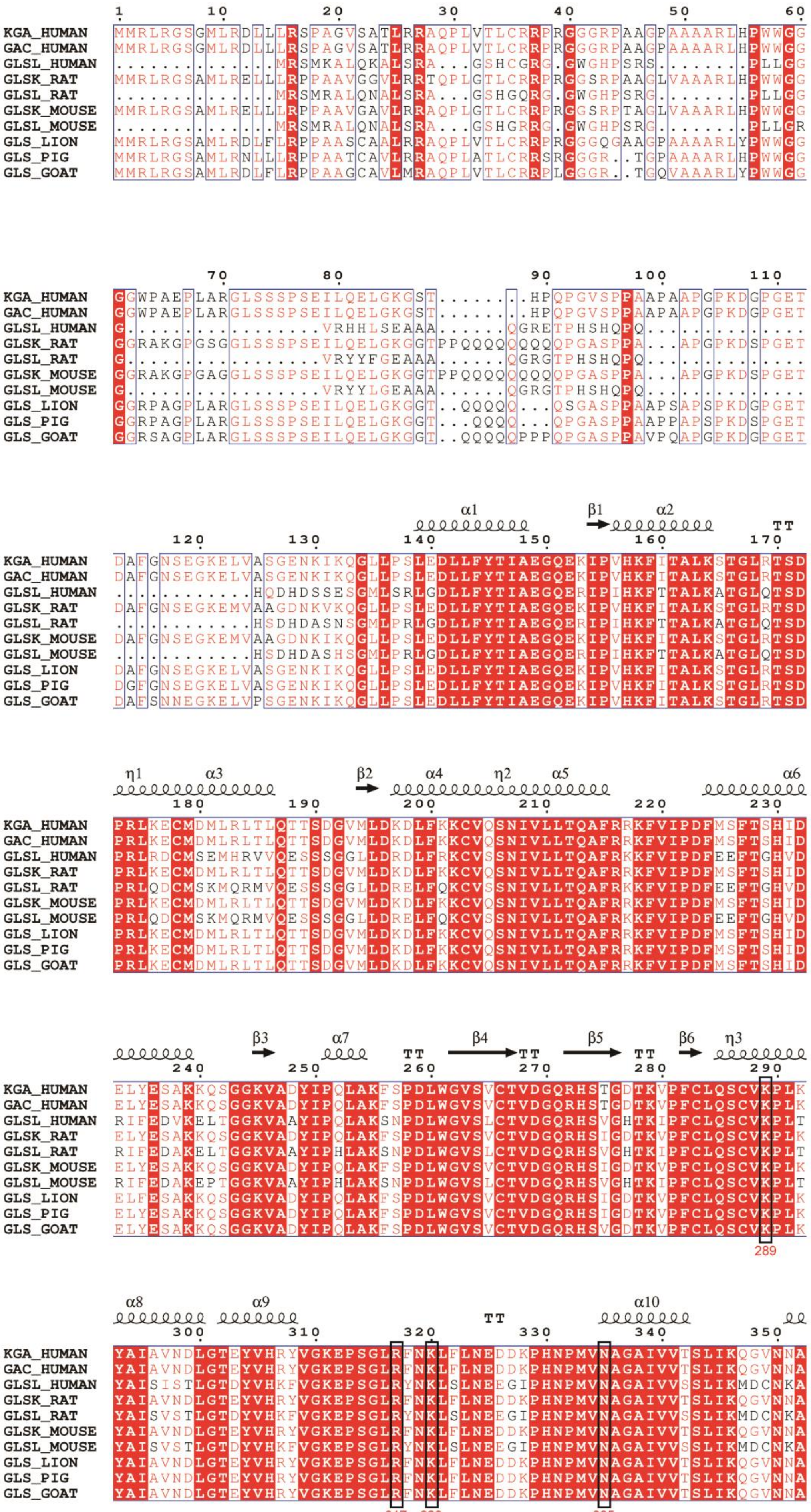
1 10 20 30 40 50 60
KGA_HUMAN MMRLRGSGMLRDLLLRSPAGVSATLRRAQPLVTLCRRPRGGGRPAAGPAAAARLHPWWGG
GAC_HUMAN MMRLRGSGMLRDLLLRSPAGVSATLRRAQPLVTLCRRPRGGGRPAAGPAAAARLHPWWGG
GLSL_HUMAN .............MRSMKALQKALSRA...GSHCGRG.GWGHPSRS.......PLLGG
GLSK_RAT MMRLRGSAMLRELLLRPPAAVGGVLRRTQPLGTLCRRPRGGSRPAAGLVAAARLHPWWGG
GLSL_RAT .............MRSMRALQNALSRA...GSHGQRG.GWGHPSRG.......PLLGG
GLSK_MOUSE MMRLRGSAMLRELLLRPPAAVGAVLRRAQPLGTLCRRPRGGSRPTAGLVAAARLHPWWGG
GLSL_MOUSE .............MRSMRALQNALSRA...GSHGRRG.GWGHPSRG.......PLLGR
GLS_LION MMRLRGSAMLRDLFLRPPAASCAALRRAQPLVTLCRRPRGGGQGAAGPAAAARLYPWWGG
GLS_PIG MMRLRGSAMLRNLLLRPPAATCAVLRRAQPLATLCRRSRGGGR..TGPAAAARLHPWWGG
GLS_GOAT MMRLRGSAMLRDLFLRPPAAGCAVLMRAQPLVTLCRRPLGGGR..TGQVAAARLYPWWGG
70 80 90 100 110
KGA_HUMAN GGWPAEPLARGLSSSPSEILQELGKGST........HPQPGVSPPAAPAAPGPKDGPGET
GAC_HUMAN GGWPAEPLARGLSSSPSEILQELGKGST........HPQPGVSPPAAPAAPGPKDGPGET
GLSL_HUMAN G..............VRHHLSEAAA......QGRETPHSHQPQ.................
GLSK_RAT GGRAKGPGSGGLSSSPSEILQELGKGGTPPQQQQQQQQQPGASPPA...APGPKDSPGET
GLSL_RAT G..............VRYYFGEAAA......QGRGTPHSHQPQ.................
GLSK_MOUSE GGRAKGPGAGGLSSSPSEILQELGKGGTPPQQQQQQQQQPGASPPA...APGPKDSPGET
GLSL_MOUSE G..............VRYYLGEAAA......QGRGTPHSHQPQ.................
GLS_LION GGRPAGPLARGLSSSPSEILQELGKGGT..QQQQQ...QSGASPPAAPSAPSPKDGPGET
GLS_PIG GGRPAGPLARGLSSSPSEILQELGKGGT...QQQQ...QPGASPPAAPPAPSPKDSPGET
GLS_GOAT GGRSAGPLARGLSSSPSEILQELGKGGT..QQQQQPPPQPGASPPAVPQAPGPKDGPGET
α1 β1 α2 TT
120 130 140 150 160 170
KGA_HUMAN DAFGNSEGKELVASGENKIKQGLLPSLEDLLFYTIAEGQEKIPVHKFITALKSTGLRTSD
GAC_HUMAN DAFGNSEGKELVASGENKIKQGLLPSLEDLLFYTIAEGQEKIPVHKFITALKSTGLRTSD
GLSL_HUMAN ............HQDHDSSESGMLSRLGDLLFYTIAEGQERIPIHKFTTALKATGLQTSD
GLSK_RAT DAFGNSEGKEMVAAGDNKVKQGLLPSLEDLLFYTIAEGQEKIPVHKFITALKSTGLRTSD
GLSL_RAT ............HSDHDASNSGMLPRLGDLLFYTIAEGQERIPIHKFTTALKATGLQTSD
GLSK_MOUSE DAFGNSEGKEMVAAGDNKIKQGLLPSLEDLLFYTIAEGQEKIPVHKFITALKSTGLRTSD
GLSL_MOUSE ............HSDHDASHSGMLPRLGDLLFYTIAEGQERIPIHKFTTALKATGLQTSD
GLS_LION DAFGNSEGKELVASGENKIKQGLLPSLEDLLFYTIAEGQEKIPVHKFITALKSTGLRTSD
GLS_PIG DGFGNSEGKELVASGENKIKQGLLPSLEDLLFYTIAEGQEKIPVHKFITALKSTGLRTSD
GLS_GOAT DAFSNNEGKELVPSGENKIKQGLLPSLEDLLFYTIAEGQEKIPVHKFITALKSTGLRTSD
η1 α3 β2 α4 η2 α5 α6
180 190 200 210 220 230
KGA_HUMAN PRLKECMDMLRLTLQTTSDGVMLDKDLFKKCVQSNIVLLTQAFRRKFVIPDFMSFTSHID
GAC_HUMAN PRLKECMDMLRLTLQTTSDGVMLDKDLFKKCVQSNIVLLTQAFRRKFVIPDFMSFTSHID
GLSL_HUMAN PRLRDCMSEMHRVVQESSSGGLLDRDLFRKCVSSNIVLLTQAFRKKFVIPDFEEFTGHVD
GLSK_RAT PRLKECMDMLRLTLQTTSDGVMLDKDLFKKCVQSNIVLLTQAFRRKFVIPDFMSFTSHID
GLSL_RAT PRLQDCMSKMQRMVQESSSGGLLDRELFQKCVSSNIVLLTQAFRKKFVIPDFEEFTGHVD
GLSK_MOUSE PRLKECMDMLRLTLQTTSDGVMLDKDLFKKCVQSNIVLLTQAFRRKFVIPDFMSFTSHID
GLSL_MOUSE PRLQDCMSKMQRMVQESSSGGLLDRELFQKCVSSNIVLLTQAFRKKFVIPDFEEFTGHVD
GLS_LION PRLKECMDMLRLTLQTTSDGVMLDKDLFKKCVQSNIVLLTQAFRRKFVIPDFMSFTSHID
GLS_PIG PRLKECMDMLRLTLQTTSDGVMLDKDLFKKCVQSNIVLLTQAFRRKFVIPDFMSFTSHID
GLS_GOAT PRLKECMDMLRLTLQTTSDGVMLDKDLFKKCVQSNIVLLTQAFRRKFVIPDFMSFTSHID
β3 α7 TT β4 TT β5 TT β6 η3
240 250 260 270 280 290
KGA_HUMAN ELYESAKKQSGGKVADYIPQLAKFSPDLWGVSVCTVDGQRHSTGDTKVPFCLQSCVKPLK
GAC_HUMAN ELYESAKKQSGGKVADYIPQLAKFSPDLWGVSVCTVDGQRHSTGDTKVPFCLQSCVKPLK
GLSL_HUMAN RIFEDVKELTGGKVAAYIPQLAKSNPDLWGVSLCTVDGQRHSVGHTKIPFCLQSCVKPLT
GLSK_RAT ELYESAKKQSGGKVADYIPQLAKFSPDLWGVSVCTVDGQRHSIGDTKVPFCLQSCVKPLK
GLSL_RAT RIFEDAKELTGGKVAAYIPHLAKSNPDLWGVSLCTVDGQRHSVGHTKIPFCLQSCVKPLT
GLSK_MOUSE ELYESAKKQSGGKVADYIPQLAKFSPDLWGVSVCTVDGQRHSIGDTKVPFCLQSCVKPLK
GLSL_MOUSE RIFEDAKEPTGGKVAAYIPHLAKSNPDLWGVSLCTVDGQRHSVGHTKIPFCLQSCVKPLT
GLS_LION ELFESAKKQSGGKVADYIPQLAKFSPDLWGVSVCTVDGQRHSIGDTKVPFCLQSCVKPLK
GLS_PIG ELYESAKKQSGGKVADYIPQLAKFSPDLWGVSVCTVDGQRHSIGDTKVPFCLQSCVKPLK
GLS_GOAT ELYESAKKQSGGKVADYIPQLAKFSPDLWGVSVCTVDGQRHSVGDTKVPFCLQSCVKPLK
289
α8 α9 TT α10
300 310 320 330 340 350
KGA_HUMAN YAIAVNDLGTEYVHRYVGKEPSGLRFNKLFLNEDDKPHNPMVNAGAIVVTSLIKQGVNNA
GAC_HUMAN YAIAVNDLGTEYVHRYVGKEPSGLRFNKLFLNEDDKPHNPMVNAGAIVVTSLIKQGVNNA
GLSL_HUMAN YAISISTLGTDYVHKFVGKEPSGLRYNKLSLNEEGIPHNPMVNAGAIVVSSLIKMDCNKA
GLSK_RAT YAIAVNDLGTEYVHRYVGKEPSGLRFNKLFLNEDDKPHNPMVNAGAIVVTSLIKQGVNNA
GLSL_RAT YAISVSTLGTDYVHKFVGKEPSGLRYNKLSLNEEGIPHNPMVNAGAIVVSSLIKMDCNKA
GLSK_MOUSE YAIAVNDLGTEYVHRYVGKEPSGLRFNKLFLNEDDKPHNPMVNAGAIVVTSLIKQGVNNA
GLSL_MOUSE YAISVSTLGTDYVHKFVGKEPSGLRYNKLSLNEEGIPHNPMVNAGAIVVSSLIKMDCNKA
GLS_LION YAIAVNDLGTEYVHRYVGKEPSGLRFNKLFLNEDDKPHNPMVNAGAIVVTSLIKQGVNNA
GLS_PIG YAIAVNDLGTEYVHRYVGKEPSGLRFNKLFLNEDDKPHNPMVNAGAIVVTSLIKQGVNNA
GLS_GOAT YAIAVNDLGTEYVHRYVGKEPSGLRFNKLFLNEDDKPHNPMVNAGAIVVTSLIKQGVNNA
317 320 335

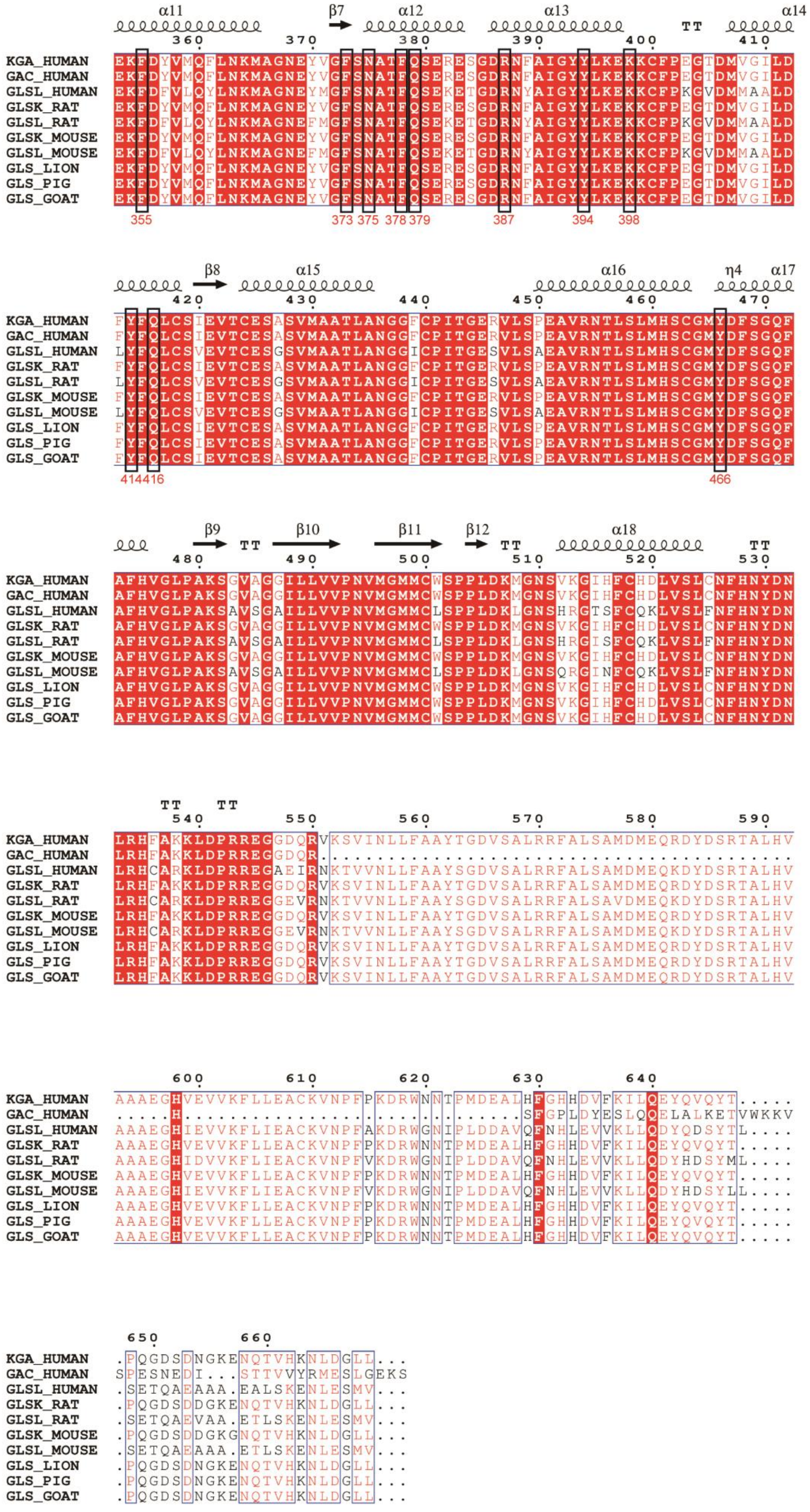
α11 β7 α12 α13 TT α14
360 370 380 390 400 410
KGA_HUMAN EKFDYVMQFLNKMAGNEYVGFSNATFQSERESGDRNFAIGYYLKEKKCFPEGTDMVGILD
GAC_HUMAN EKFDYVMQFLNKMAGNEYVGFSNATFQSERESGDRNFAIGYYLKEKKCFPEGTDMVGILD
GLSL_HUMAN EKFDFVLQYLNKMAGNEYMGFSNATFQSEKETGDRNYAIGYYLKEKKCFPKGVDMMAALD
GLSK_RAT EKFDYVMQFLNKMAGNEYVGFSNATFQSERESGDRNFAIGYYLKEKKCFPEGTDMVGILD
GLSL_RAT EKFDFVLQYLNKMAGNEFMGFSNATFQSEKETGDRNYAIGYYLKEKKCFPKGVDMMAALD
GLSK_MOUSE EKFDYVMQFLNKMAGNEYVGFSNATFQSERESGDRNFAIGYYLKEKKCFPEGTDMVGILD
GLSL_MOUSE EKFDFVLQYLNKMAGNEFMGFSNATFQSEKETGDRNYAIGYYLKEKKCFPKGVDMMAALD
GLS_LION EKFDYVMQFLNKMAGNEYVGFSNATFQSERESGDRNFAIGYYLKEKKCFPEGTDMVGILD
GLS_PIG EKFDYVMQFLNKMAGNEYVGFSNATFQSERESGDRNFAIGYYLKEKKCFPEGTDMVGILD
GLS_GOAT EKFDYVMQFLNKMAGNEYVGFSNATFQSERESGDRNFAIGYYLKEKKCFPEGTDMVGILD
355 373 375 378 379 387 394 398
β8 α15 α16 η4 α17
420 430 440 450 460 470
KGA_HUMAN FYFQLCSIEVTCESASVMAATLANGGFCPITGERVLSPEAVRNTLSLMHSCGMYDFSGQF
GAC_HUMAN FYFQLCSIEVTCESASVMAATLANGGFCPITGERVLSPEAVRNTLSLMHSCGMYDFSGQF
GLSL_HUMAN LYFQLCSVEVTCESGSVMAATLANGGICPITGESVLSAEAVRNTLSLMHSCGMYDFSGQF
GLSK_RAT FYFQLCSIEVTCESASVMAATLANGGFCPITGERVLSPEAVRNTLSLMHSCGMYDFSGQF
GLSL_RAT LYFQLCSVEVTCESGSVMAATLANGGICPITGESVLSAEAVRNTLSLMHSCGMYDFSGQF
GLSK_MOUSE FYFQLCSIEVTCESASVMAATLANGGFCPITGERVLSPEAVRNTLSLMHSCGMYDFSGQF
GLSL_MOUSE LYFQLCSVEVTCESGSVMAATLANGGICPITGESVLSAEAVRNTLSLMHSCGMYDFSGQF
GLS_LION FYFQLCSIEVTCESASVMAATLANGGFCPITGERVLSPEAVRNTLSLMHSCGMYDFSGQF
GLS_PIG FYFQLCSIEVTCESASVMAATLANGGFCPITGERVLSPEAVRNTLSLMHSCGMYDFSGQF
GLS_GOAT FYFQLCSIEVTCESASVMAATLANGGFCPITGERVLSPEAVRNTLSLMHSCGMYDFSGQF
414416 466
β9 TT β10 β11 β12 TT α18 TT
480 490 500 510 520 530
KGA_HUMAN AFHVGLPAKSGVAGGILLVVPNVMGMMCWSPPLDKMGNSVKGIHFCHDLVSLCNFHNYDN
GAC_HUMAN AFHVGLPAKSGVAGGILLVVPNVMGMMCWSPPLDKMGNSVKGIHFCHDLVSLCNFHNYDN
GLSL_HUMAN AFHVGLPAKSAVSGAILLVVPNVMGMMCLSPPLDKLGNSHRGTSFCQKLVSLFNFHNYDN
GLSK_RAT AFHVGLPAKSGVAGGILLVVPNVMGMMCWSPPLDKMGNSVKGIHFCHDLVSLCNFHNYDN
GLSL_RAT AFHVGLPAKSAVSGAILLVVPNVMGMMCLSPPLDKLGNSHRGISFCQKLVSLFNFHNYDN
GLSK_MOUSE AFHVGLPAKSGVAGGILLVVPNVMGMMCWSPPLDKMGNSVKGIHFCHDLVSLCNFHNYDN
GLSL_MOUSE AFHVGLPAKSAVSGAILLVVPNVMGMMCLSPPLDKLGNSQRGINFCQKLVSLFNFHNYDN
GLS_LION AFHVGLPAKSGVAGGILLVVPNVMGMMCWSPPLDKMGNSVKGIHFCHDLVSLCNFHNYDN
GLS_PIG AFHVGLPAKSGVAGGILLVVPNVMGMMCWSPPLDKMGNSVKGIHFCHDLVSLCNFHNYDN
GLS_GOAT AFHVGLPAKSGVAGGILLVVPNVMGMMCWSPPLDKMGNSVKGIHFCHDLVSLCNFHNYDN
TT TT
540 550 560 570 580 590
KGA_HUMAN LRHFAKKLDPRREGGDQRVKSVINLLFAAYTGDVSALRRFALSAMDMEQRDYDSRTALHV
GAC_HUMAN LRHFAKKLDPRREGGDQR..
GLSL_HUMAN LRHCARKLDPRREGAEIRNKTVVNLLFAAYSGDVSALRRFALSAMDMEQKDYDSRTALHV
GLSK_RAT LRHFAKKLDPRREGGDQRVKSVINLLFAAYTGDVSALRRFALSAMDMEQRDYDSRTALHV
GLSL_RAT LRHCARKLDPRREGGEVRNKTVVNLLFAAYSGDVSALRRFALSAVDMEQKDYDSRTALHV
GLSK_MOUSE LRHFAKKLDPRREGGDQRVKSVINLLFAAYTGDVSALRRFALSAMDMEQRDYDSRTALHV
GLSL_MOUSE LRHCARKLDPRREGGEVRNKTVVNLLFAAYSGDVSALRRFALSAMDMEQKDYDSRTALHV
GLS_LION LRHFAKKLDPRREGGDQRVKSVINLLFAAYTGDVSALRRFALSAMDMEQRDYDSRTALHV
GLS_PIG LRHFAKKLDPRREGGDQRVKSVINLLFAAYTGDVSALRRFALSAMDMEQRDYDSRTALHV
GLS_GOAT LRHFAKKLDPRREGGDQRVKSVINLLFAAYTGDVSALRRFALSAMDMEQRDYDSRTALHV
600 610 620 630 640
KGA_HUMAN AAAEGHVEVVKFLLEACKVNPFPKDRWNNTPMDEALHFGHHDVFKILQEYQVQYT.....
GAC_HUMAN .....H..............................SFGPLDYESLQQELALKETVWKKV
GLSL_HUMAN AAAEGHIEVVKFLIEACKVNPFAKDRWGNIPLDDAVQFNHLEVVKLLQDYQDSYTL....
GLSK_RAT AAAEGHVEVVKFLLEACKVNPFPKDRWNNTPMDEALHFGHHDVFKILQEYQVQYT.....
GLSL_RAT AAAEGHIDVVKFLIEACKVNPFVKDRWGNIPLDDAVQFNHLEVVKLLQDYHDSYML....
GLSK_MOUSE AAAEGHVEVVKFLLEACKVNPFPKDRWNNTPMDEALHFGHHDVFKILQEYQVQYT.....
GLSL_MOUSE AAAEGHIEVVKFLIEACKVNPFVKDRWGNIPLDDAVQFNHLEVVKLLQDYHDSYLL....
GLS_LION AAAEGHVEVVKFLLEACKVNPFPKDRWNNTPMDEALHFGHHDVFKILQEYQVQYT.....
GLS_PIG AAAEGHVEVVKFLLEACKVNPFPKDRWNNTPMDEALHFGHHDVFKILQEYQVQYT.....
GLS_GOAT AAAEGHVEVVKFLLEACKVNPFPKDRWNNTPMDEALHFGHHDVFKILQEYQVQYT.....
650 660
KGA_HUMAN .PQGDSDNGKENQTVHKNLDGLL...
GAC_HUMAN SPESNEDI...STTVVYRMESLGEKS
GLSL_HUMAN .SETQAEAAA.EALSKENLESMV...
GLSK_RAT .PQGDSDDGKENQTVHKNLDGLL...
GLSL_RAT .SETQAEVAA.ETLSKENLESMV...
GLSK_MOUSE .PQGDSDDGKGNQTVHKNLDGLL...
GLSL_MOUSE .SETQAEAAA.ETLSKENLESMV...
GLS_LION .PQGDSDNGKENQTVHKNLDGLL...
GLS_PIG .PQGDSDNGKENQTVHKNLDGLL...
GLS_GOAT .PQGDSDNGKENQTVHKNLDGLL...

Fig. S5. Sequence alignment of GLS from different mammalian species. The sequence alignment of the amino acid sequence of human KGA and GAC, human GLSL, rat GLSK, rat GLSL, mouse GLSK, mouse GLSL, lion GLS, pig GLS, and goat GLS is shown. The conserved residues are shaded in red, and secondary structure information is indicated above the alignment.

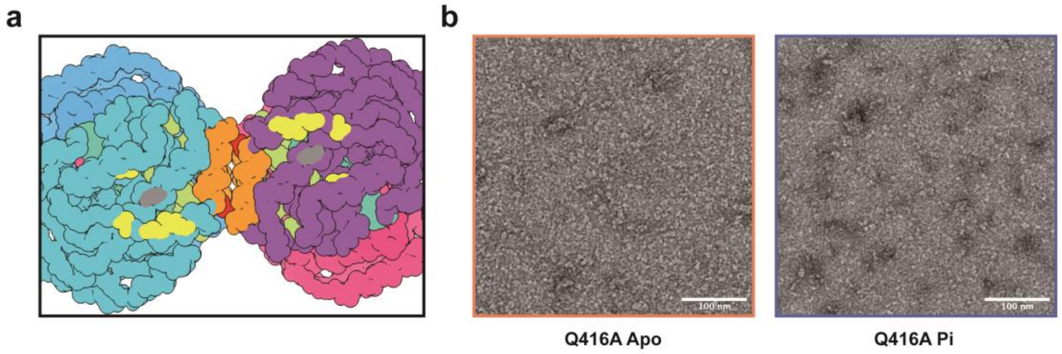

Fig. S6. Mutation of filament interface residues.

a) Q416 locates on the center of GAC helical interface. GAC tetramer is colored as Fig 1h. Residues of the helical interface are colored in yellow and Q416 in grey. **b)** Negative stain electron microscopy micrographs of Q416A^{Apo} and Q416A^{Pi} incubated at 37 °C for 10 min.

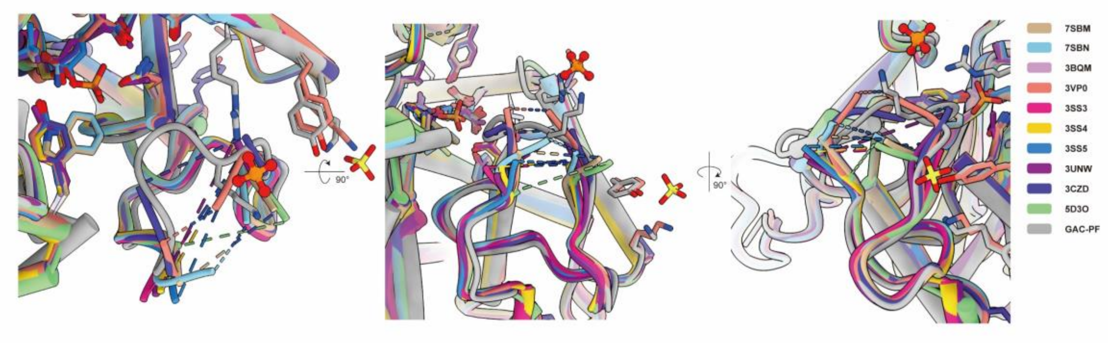

Fig. S7. High flexibility of AL in mammalian glutaminase model without inhibitor binding.

Comparison of the AL in GAC-PF with different models. The disordered loop is indicated by dashes. The AL in other models is shown to be highly flexible, suggesting its potential role in regulating the enzyme activity.

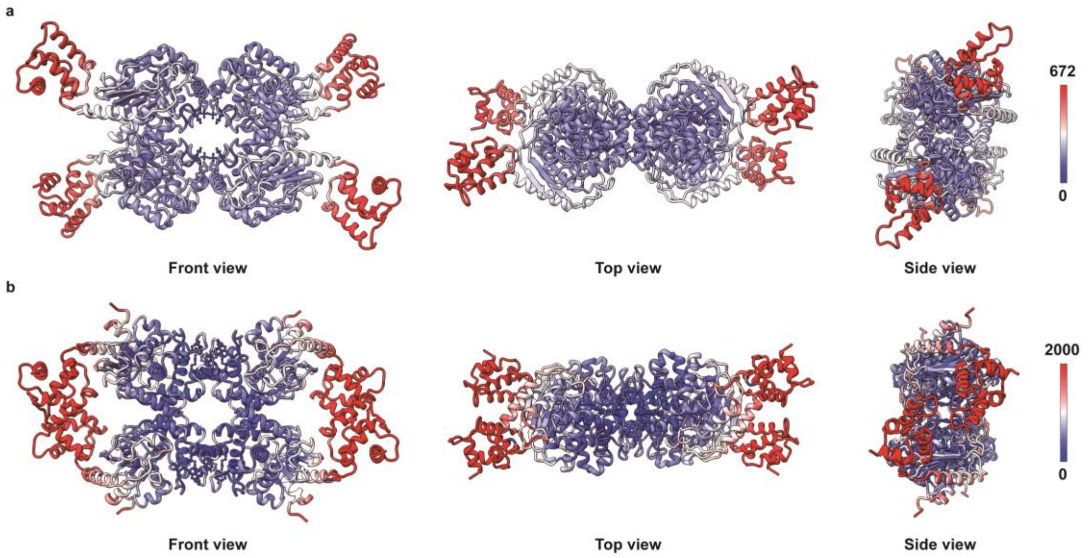

Fig. S8. B-factor analysis of GAC-PF model.

a) B-factor analysis of the helical unit model, with the color gradient representing the B-factor values ranging from low (blue) to high (red). **b)** B-factor analysis of the helical interface, with the color gradient representing the B-factor values ranging from low (blue) to high (red).

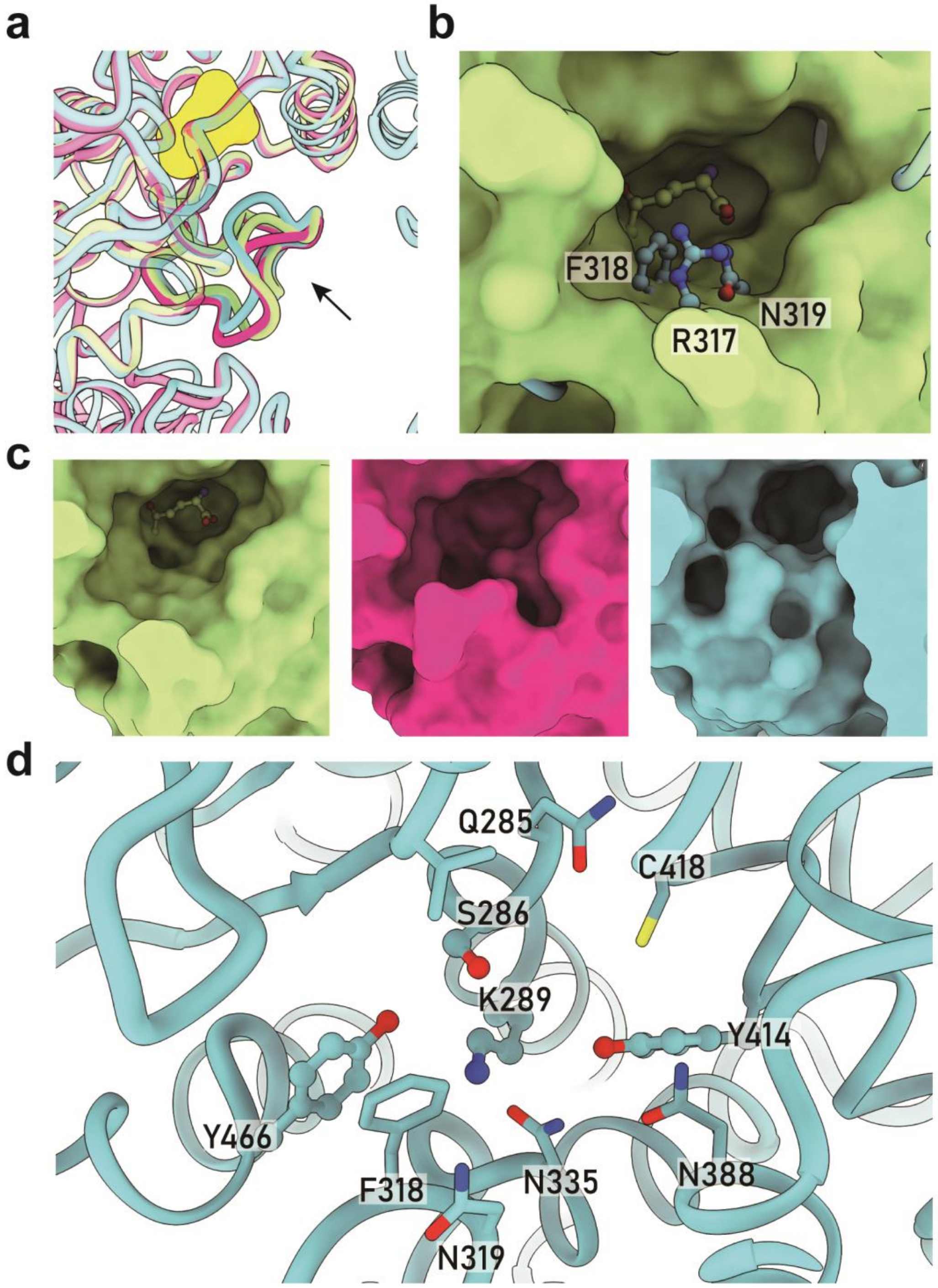

Fig. S9. Filamentation by Pi remodels the catalytic pocket of glutaminase.

a) The activation loop (AL) region in GAC-PF tetramer is reshaped towards the catalytic center which is displayed by yellow surface, compared to AL obtained in other models (PDB ID: 4O7D and 5W2J). **b)**

The reshaped AL further fills the catalytic pocket, as evidenced by the green surface of the 5W2J model being filled with residues R317, F318, and N319 from the AL of GAC-PF. **c)** Pocket surface comparison. **d)** The detailed composition of the remodeled catalytic pocket of glutaminase is shown with the density map.

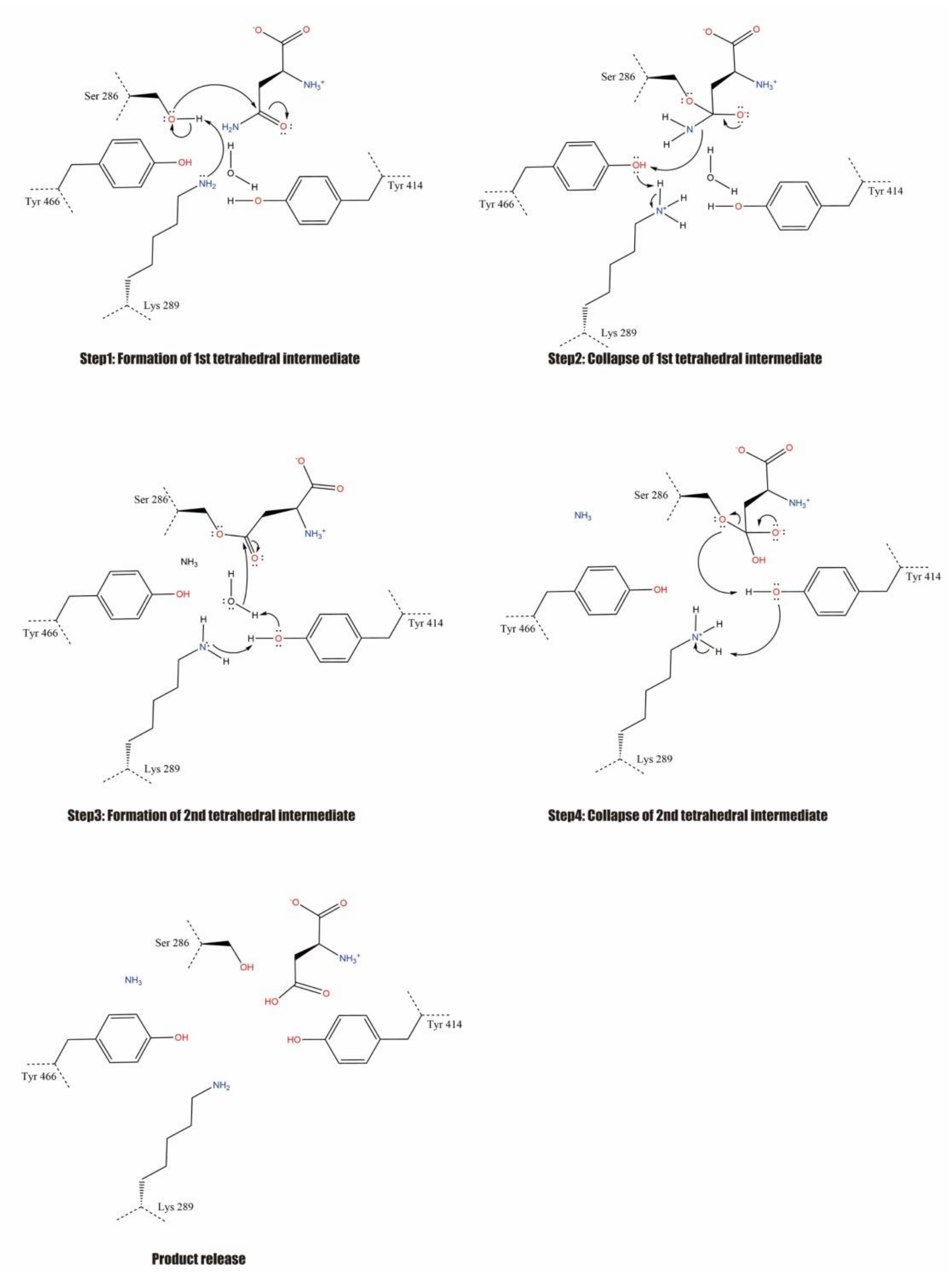

Fig. S10. Proposed catalytic diagram for glutaminase.

The catalytic reaction of GAC can be roughly divided into four steps: 1) K289 deprotonates S286, initiating a nucleophilic addition onto the carbonyl carbon of the substrate amide group, forming the first tetrahedral intermediate; 2) The first tetrahedral intermediate collapses, cleaving the

C-N bond and releasing the ammonia product, the nitrogen of which deprotonates K289 via a proton relay through Y466; 3) K289 deprotonates water via a proton relay with Y414, initiating a nucleophilic addition at the carbonyl carbon, forming a new tetrahedral intermediate; 4) The new tetrahedral intermediate collapses, cleaving the acyl-enzyme bond and liberating S286, which in turn deprotonates K289 via a proton relay with Y414.

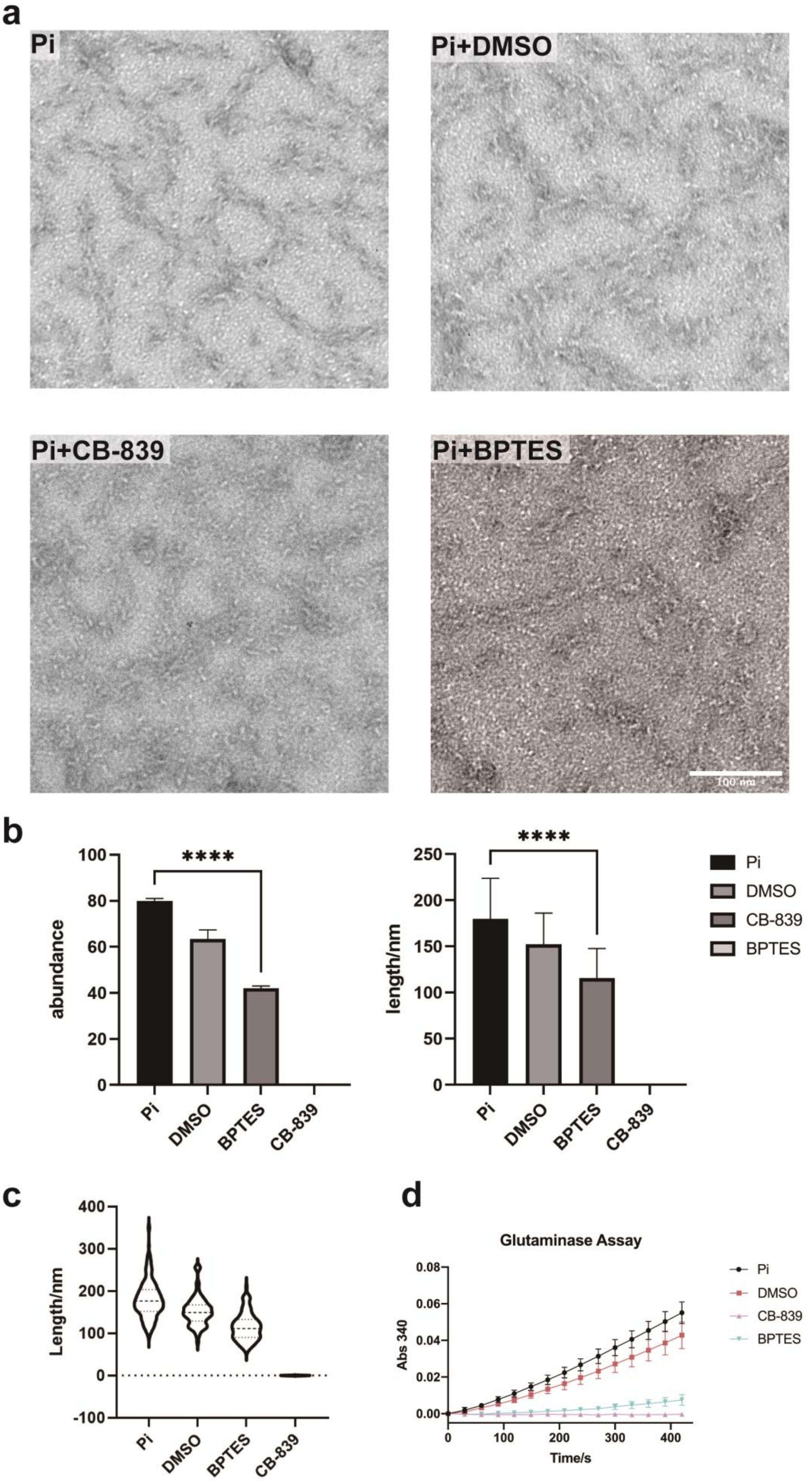
a
Pi
Pi+DMSO
Pi+CB-839
Pi+BPTES
100 nm
b
abundance
****
Pi
DMSO
BPTES
CB-839
length/nm
****
Pi
DMSO
CB-839
BPTES
c
Length/nm
Pi
DMSO
BPTES
CB-839
d
Glutaminase Assay
Abs 340
Time/s
Pi
DMSO
CB-839
BPTES

Fig. S11. Negative staining analysis and glutaminase assay for inhibitor joined sample.

a) Negative stain electron microscopy micrographs of GAC^{Pi} with or without inhibitors. Fewer filaments were observed after incubating with inhibitors BPTES or CB-839, with DMSO as a negative control. Scale bar represents 100 nm. **b)** Quantification of negative staining images showing the disruption of GAC filamentation by BPTES and CB-839. **c)** The distribution of GAC filament length. **d)** Inhibition of GAC by BPTES or CB-839 shown by glutaminase assay for GAC^{Pi} with or without inhibitors, and DMSO as a control.

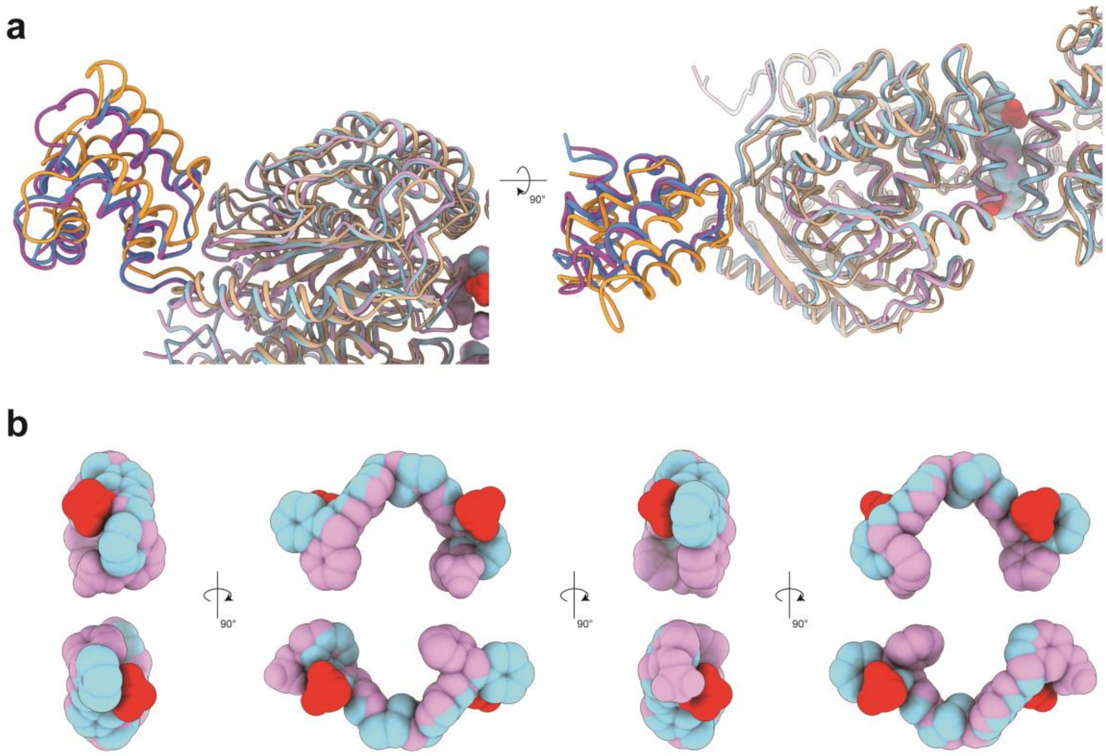

Fig. S12. Structures comparison of GAC-PF and inhibitors bound GAC.

a) A zoom-in view of the conformation changes of the GAC tetramer induced by BPTES or CB-839 binding. The N termini of each model is colored differently to the catalytical core. **b)** Detailed view of the overlap between Pi and inhibitors.

Supplementary Table S1. Cryo-EM data collection and model refinement.

	helical interface (EMD-35574, PDB 8IMB)	helical unit (EMD-35573, PDB 8IMA)
**Data collection**		
EM equipment	Titan Krios	Titan Krios
Detector	K3 camera	K3 camera
Magnification	22,500x	22,500x
Voltage (kV)	300	300
Electron exposure ((e–/Å^2))	50	50
Defocus range(μm)	-1.2 to -1.8	-1.2 to -1.8
Pixel size(Å)	1.06	1.06
Symmetry imposed	D2	D2
Number of collected movies	9265	9265
Initial particle images (no.)	3271231	3271231
Final particle images (no.)	321414	203004
Map resolution (Å)	2.9	2.9
FSC threshold	0.143	0.143
Map resolution range (Å)	2.8-3.8	2.8-3.7
**Refinement**		
Initial model used (PDB code)	3SS4	3SS4
Map sharpening B-factor(Å^2)	80	80
Model composition		
Non-hydrogen atoms	12304	12304
Protein residues	1580	1580
Ligands	PO4: 4	PO4: 4
Waters	0	0
B factors(Å^2)	80	80
R.m.s. deviations		
Bond lengths (Å)	0.004	0.004
Bond angles (°)	0.564	0.54
Validation		
MolProbity score	1.83	2.37
Clashscore	4.25	9.97
Poor rotamers (%)	1.75	2.92
Ramachandran plot		
Favored (%)	93.19	91.79
Allowed (%)	6.81	8.21
Disallowed (%)	0	0
